# Trends of musculoskeletal pain in children and young people consulting primary care: an electronic primary health care record study

**DOI:** 10.1186/s12887-025-06296-y

**Published:** 2025-11-25

**Authors:** Kayleigh J. Mason, Kelvin P. Jordan, James Bailey, Joanne Protheroe, Faraz Mughal, Martin J. Thomas, Anna Saxne Jöud, Sue Jowett, Kym I.E. Snell, Kate M. Dunn

**Affiliations:** 1 Primary Care Centre Versus Arthritis, School of Medicine, Keele University, Keele, United Kingdom; 2 Haywood Academic Rheumatology Centre, Midlands Partnership University NHS Foundation Trust, Haywood Hospital, Stoke-on-Trent, United Kingdom; 3 Epidemiology for Health, Division of Occupational and Environmental Medicine, Lund University, Lund, Sweden; 4 Department of Applied Health Research, School of Health Sciences, College of Medicine and Health, University of Birmingham, Birmingham, United Kingdom

**Keywords:** Pediatrics, Epidemiology, Primary health care, Musculoskeletal pain, Children, Young people

## Abstract

**Background:**

Pain in childhood is common, but there is an information gap on initial care seeking. Our aim was to determine trends and variations in the consultation prevalence and incidence of musculoskeletal pain, and most common sites of pain, in children and young people presenting to UK primary care.

**Methods:**

A national UK primary care database (CPRD Aurum) was used to determine annual prevalence and incidence of consultation for musculoskeletal pain in children and young people (aged 8–18 years) between 2005 and 2021. Incidence was defined as no musculoskeletal consultation in the previous 12 months. Rates were calculated per 10,000 registered population for each calendar year and by age, gender, and body site.

**Results:**

1,175,641 children and young people (49% female; median age 12) consulted primary care for musculoskeletal pain. Annual consultation prevalence for musculoskeletal pain rose from 808/10,000 in 2005 to 980/10,000 in 2011, then remained stable to 2015 before falling slightly. Annual consultation rates were higher for younger and older females, although minimal differences were observed between genders for 12–15-year-olds. Foot/ankle was the most common pain site in younger children. Back pain was the most recorded pain site for females from age 16.

**Conclusions:**

This study provides contemporary data on how common consultations for musculoskeletal pain are in children and young people using nationally representative primary care electronic health records. It demonstrates that musculoskeletal pain is common in children and young people with nearly one in ten seeking primary health care each year, with different patterns by age, gender and body site. Understanding the epidemiology of musculoskeletal pain in children and young people presenting to primary care is key due to paucity of information on how to effectively care for this population and highlights a need to ensure that service provision is adapted accordingly.

**Supplementary Information:**

The online version contains supplementary material available at 10.1186/s12887-025-06296-y.

## Introduction

Musculoskeletal pain is a leading cause of disability worldwide [1]. Whilst common in adults with up to one in five presenting to general practice each year with a musculoskeletal condition [2], musculoskeletal pain is present throughout the life course [3]. For example, observational studies have reported over half of 9 year-olds report pain in the last month [4], and up to 44% of children and young people report chronic pain [5, 6]. Back and neck pain are among the top 10 causes of disability globally among 10–14 year-olds, rising to fourth place among 15–19 year-olds, before becoming the leading cause among 25–29 year-olds [1]. 

There is evidence that pain in childhood is related to musculoskeletal pain among adults [7–9] but there is an absence of musculoskeletal pain research in primary care among children and young people at population level [10–12], with limited evidence on the types of musculoskeletal pain presentations in this setting and how they vary by demographics such as age and gender. In the UK, nearly everyone is registered with primary care, and so primary healthcare use reflects population level healthcare seeking. Understanding the healthcare seeking behaviours of children and young people with musculoskeletal pain is important because primary care is the setting where most musculoskeletal problems are identified and managed, and provides a point at which early intervention can effect lasting change.

The aim of this study was to determine trends in the prevalence and incidence of primary care consultations for musculoskeletal pain in children and young people, and describe variation by age, gender and site of pain.

## Methods

### Study Design, setting and population

The study utilised the UK Clinical Practice Research Datalink (CPRD) Aurum, a nationally representative database of anonymised primary care electronic health records (EHR) for over 41 million patients from over 1,400 general practices using EMIS Web^®^ software, currently covering 19.8% of the UK population [10, 11]. 

#### Denominator population

Children and young people aged 8–18 years with at least 24 months prior registration at their general practice before 1 st January of each calendar year (2005–2021) and a registration end date after the 31 st December of each year were included in the population at risk of consulting for musculoskeletal pain. Where registration end dates were missing, the earliest of the practice’s last collection date or a patient’s death date was used. Demographic data available for the population at risk are gender, year of birth, geographical region, and key dates related to general practice registration.

The study was approved by the CPRD Research Data Governance (ref 22_002318). The approved protocol was made available to reviewers.

### Prevalence of musculoskeletal consultations

#### Numerator population

Within each calendar year, children and young people who had a recorded primary care consultation for musculoskeletal pain in that year were included as prevalent musculoskeletal consulters. Each individual was counted once per year for prevalence estimates. For incidence estimates, we excluded individuals with a musculoskeletal consultation in the preceding 12 months to capture new episodes of care. In UK primary care, diagnoses and symptoms were recorded using the Read code system up to 2018 and Systematized Nomenclature of Medicine Clinical Terms (SNOMED CT) codes have been used since 2018. Free text fields in the medical records are not available to search in order to maintain patient confidentiality where identifiable information might be included in free text. Read and SNOMED CT Code lists for musculoskeletal pain were derived from published works [12, 13] and reviewed and finalised by consensus of two General Practitioners [FM; JP] and a Specialist Rheumatology and Musculoskeletal Physiotherapist [MJT]). Our definition of musculoskeletal pain consultations was broad to determine consultations for acute (e.g., trauma – sprains, strains, injuries) and chronic musculoskeletal pain (e.g., diagnoses – scoliosis, juvenile idiopathic arthritis), and were categorised by pain site (arm; back; chest/trunk; elbow; foot/ankle; hand/wrist; head; hip/pelvis; knee; leg; multiple; neck; other; shoulder). Some of the included codes may not always be associated with pain in childhood (e.g., pas planus/fallen arches, flat foot, knock-kneed) but were included as likely to be causing discomfort if recorded as a reason for consulting primary care. The final code list is available at 10.21252/c3kq-pt76.

### Statistical analysis

Annual consultation prevalence rate was calculated overall and by pain site per 10,000 registered persons for each year between 2005 and 2021. This was repeated for incidence rate, and also stratified by gender, age bands (8–11, 12–15 and 16–18 years; recommended through consultation with a Young Persons Advisory Group as ages of transition in the UK [11–12 years primary to secondary school; 15–16 years leaving secondary school]) and presented also for the 5 most common regional pain sites (back, chest/trunk, foot/ankle, hand/wrist, and knee).

Absence of a code for musculoskeletal pain will be reasonably assumed as no consultation having occurred. Individuals with missing data for year of birth (to calculate age) or gender will be excluded.

Data management and statistical analyses were performed in STATA version 17 (StataCorp, USA).

## Results

In total there were 1,175,641 children and young people who consulted primary care for musculoskeletal pain between 2005 and 2021 with 4,112,436 children and young people included in the population at risk (Table 1).

**Table 1:** Characteristics of children and young people consulting for musculoskeletal pain between 2005–2021

	Total
Number of individuals in the at-risk population, *n*	4,112,436
Number of consultations for musculoskeletal pain^a^, *n*	1,945,427
Number of individuals consulting for musculoskeletal pain	1,175,641
Median number of consultations (IQR); range	1 (1, 2); 1–11
Gender recorded on consultation, *n* (%)	
*Female*	946,257 (49)
*Male*	999,170 (51)
Median age recorded on consultation (IQR), years	12 (10, 14)
Age Bands, *n* (%)	
*8 to 11 years*	565,000 (29)
*12 to 15 years*	818,817 (42)
*16 to 18 years*	561,610 (29)

^a^ Individuals may consult more than once

The results section focuses on consultation prevalence due to the similarity in the trends observed for consultation incidence (provided in Figures S1-S3 and Tables S1a-S1c).

### Annual consultation rates

**Fig. 1:**
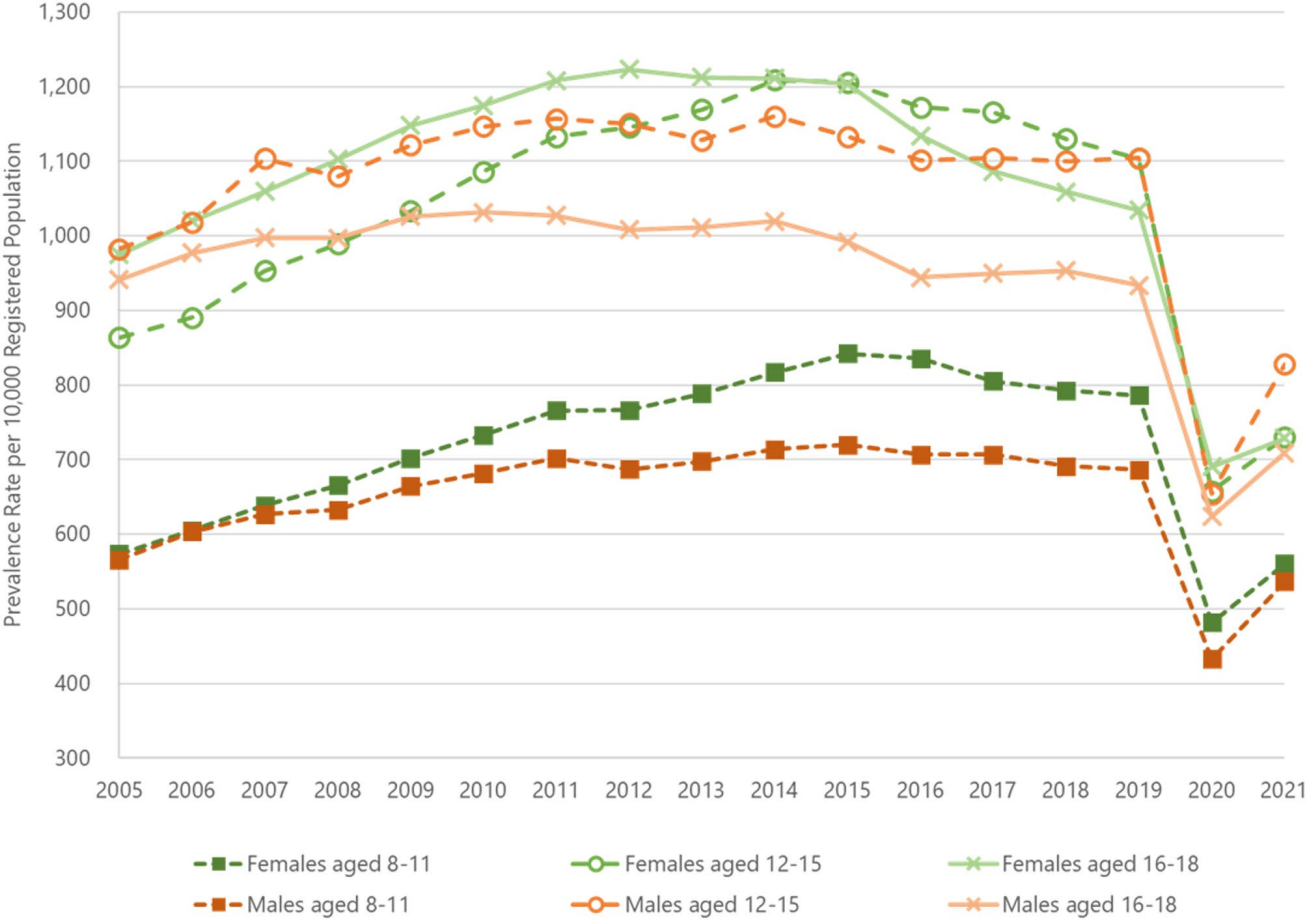
The image depicts annual prevalence rates of musculoskeletal consultations between 2005–2021 per 10,000 registered population. The rates are presented by the age groups 8–11 years (square marker, dotted lines), 12–15 years (circle marker, dashed lines) and 16–18 years (cross marker, solid lines) and by gender (females in green; males in orange)

Annual consultation prevalence for musculoskeletal pain rose from 808/10,000 in 2005 to 980/10,000 in 2011, then remained stable from 2015. Prevalence fell further to 580/10,000 in 2020 before rising to 678/10,000 in 2021 (Table S2a). Musculoskeletal pain consultation prevalence was lowest for children aged 8 to 11 years (Table S2a). Prevalence was higher in females than males in the age bands 8–11 and 16–18, but there was less of a difference by gender in those aged 12–15 years (Fig. 1 and Tables S2b-S2c). Consultation incidence rose from 699 to 849 in 2014, fell to 486 in 2020 before rising to 606 in 2021. As with prevalence, incidence was lowest in children aged 8–11 (Table S1a).

### Trends in consultations by pain site

**Fig. 2:**
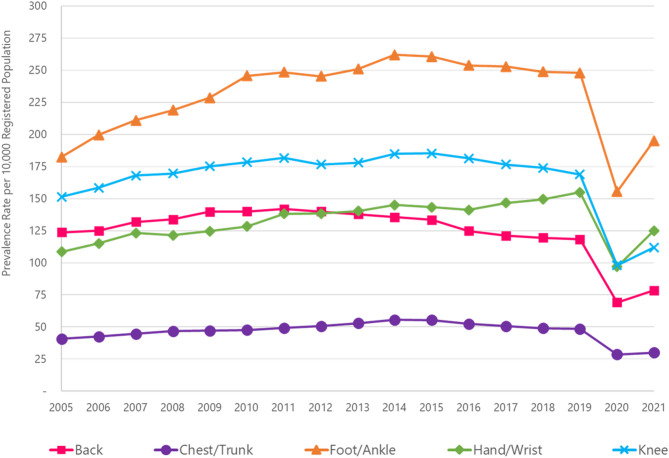
The image depicts annual prevalence rates of the most common regional pain sites per 10,000 registered population between 2005–2021. The rates are presented for back pain (square marker, pink line), chest/trunk pain (circle marker, purple line), foot/ankle pain (triangle marker, orange line), hand/wrist pain (diamond marker, green line) and knee pain (cross marker, blue line)

Patterns in trends over time were generally consistent by pain site with the foot/ankle (248/10,000 in 2019) being the most common body site for consultation overall, followed by knee (169/10,000), hand/wrist (155/10,000), back (118/10,000) and chest/trunk (48/10,000; Fig. 2 and Table S2a).

However, patterns for regional pain sites varied by age and gender. In the age group 8–11, foot/ankle consultation was more than twice as common than any other individual pain site for both males and females up to and including 2019 (Figure S4). But, in females aged 12–15, the gap was smaller with foot/ankle consultation prevalence about 20% higher than for the knee each year, whilst there was little difference between prevalence of foot/ankle and knee consultation in males. Within age group 16–18 years, the back was the most common pain site for consultation for females; in males, the knee was generally the most common pain site across each year although by 2019 there was little difference in consultation prevalence of knee, foot/ankle, back, and hand/wrist pain.

**Fig. 3:**
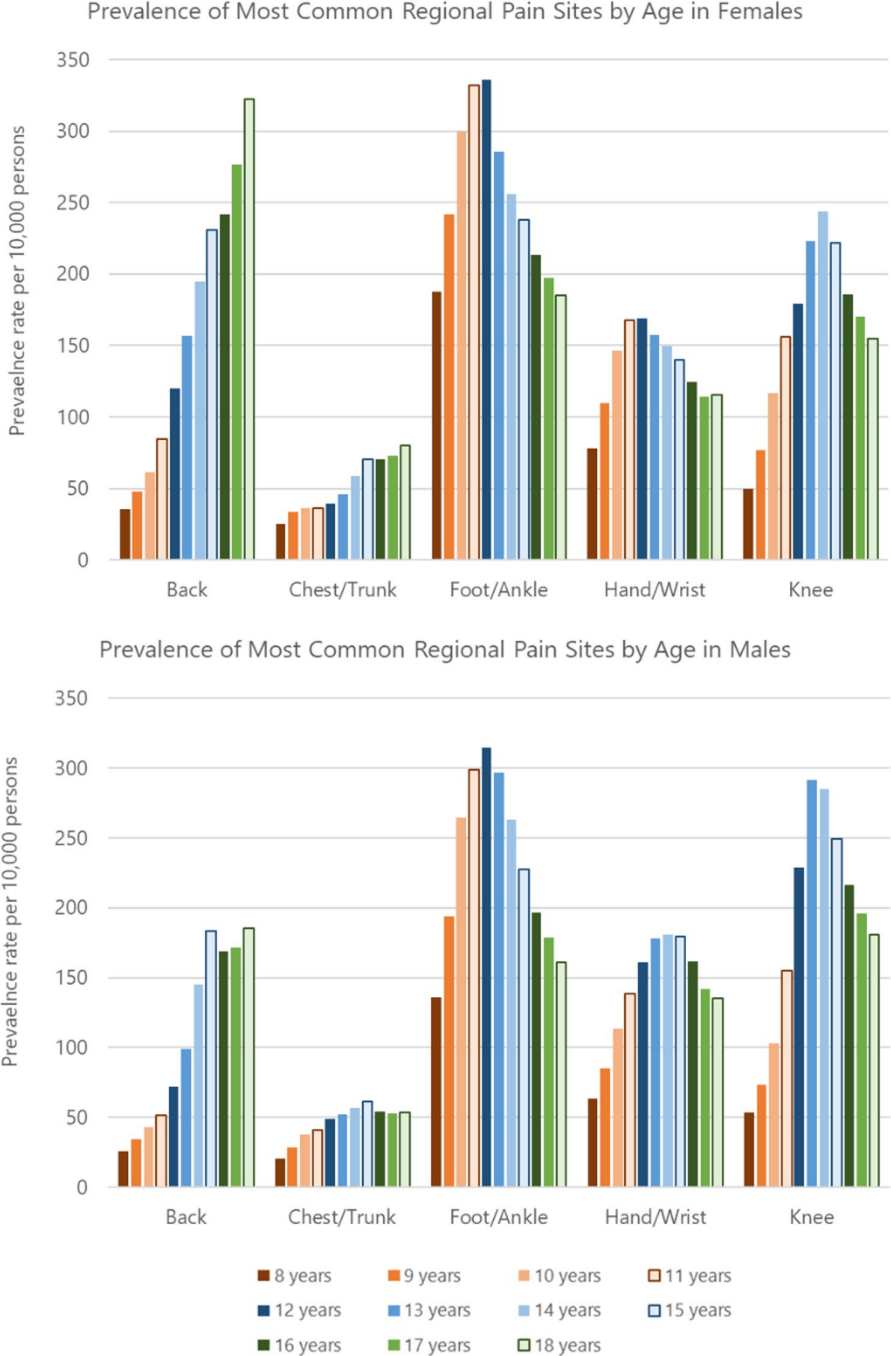
The image depicts 2 graphs with the prevalence of the most common regional pain sites by age in years and gender per 10,000 registered population. The graph for females is presented above the graph for males. Ages 8–11 are presented in shades of orange that lighten with age, with ages 12–15 presented in lightening shades of blue and ages 16–18 presented in lightening shades of green

Prevalence of foot/ankle pain peaked at 12 years and at 13–14 years for knee pain in both males and females. Peak prevalence for hand/wrist was 13–15 years for males and 11–12 years for females (Fig. 3). The prevalence of back and chest/trunk pain rose until age 18 in females while the highest rates occurred at 15 years in males (Fig. 3).

Patterns for incidence matched those for prevalence, with foot/ankle being most common site for new episodes of MSK pain in those aged 8–15, knee and foot/ankle for males aged 16–18, and back for females aged 16–18 (Figure S3).

### Types of pain

The most frequently used codes for each pain site were “*Back pain*“ and “*Low Back pain*” (33% and 32% of back codes, respectively), “*Musculoskeletal chest pain*” (40% of chest/trunk codes), “*Foot pain*” and “*Ankle sprain*” (19% and 15% of foot/ankle codes, respectively), “*Finger injury*” and “*Wrist injury”* (18% and 15% of hand/wrist codes, respectively) and “*Knee pain*”(52% of knee codes; Table 2).

**Table 2:** Top 10 codes by SNOMED CT concept ID for the most common painful body sites

Back Pain *n* = 365,646 records	Chest/Trunk Pain *n* = 115,873 records	Foot/Ankle Pain *n* = 624,047 records	Hand/Wrist Pain *n* = 349,364 records	Knee Pain *n* = 491,335 records
Back pain (*n* = 119,443; 33%)	Musculoskeletal chest pain (*n* = 45,827; 40%)	Foot pain (*n* = 116,195; 19%)	Finger injury (*n* = 63,408; 18%)	Knee pain (*n* = 256,053; 52%)
Low back pain (*n* = 118,258; 32%)	Costochondritis (*n* = 25,085; 22%)	Ankle sprain (*n* = 91,149; 15%)	Wrist injury (*n* = 52,245; 15%)	Knee injury (*n* = 53,992; 11%)
C/O - low back pain (*n* = 24,728; 7%)	Rib pain (*n* = 15,950; 14%)	Ankle pain (*n* = 67,200; 11%)	Injury of hand (*n* = 36,258; 10%)	Juvenile osteochondrosis of tibial tubercle (*n* = 53,743; 11%)
Coccygodynia (*n* = 11,245; 3%)	Chest wall pain (*n* = 8,963; 8%)	Foot injury (*n* = 49,872; 8%)	Wrist joint pain (*n* = 23,764; 7%)	Anterior knee pain (*n* = 29,201; 6%)
Idiopathic scoliosis (*n* = 7,609; 2%)	Anterior chest wall pain (*n* = 4,372; 4%)	Ankle injury (*n* = 49,730; 8%)	Wrist sprain NOS (*n* = 23,596; 7%)	Knee sprain (*n* = 17,595; 4%)
Acute thoracic back pain (*n* = 7,516; 2%)	C/O - a chest wall symptom (*n* = 3,962; 3%)	Heel pain (*n* = 34,708; 6%)	Hand pain (*n* = 20,382; 6%)	Chondromalacia patellae (*n* = 11,936; 2%)
Kyphoscoliosis and scoliosis (*n* = 7,378; 2%)	Sprain of costal cartilage (*n* = 2,439; 2%)	Flat foot (*n* = 28,670; 5%)	Thumb injury (*n* = 19,623; 6%)	Dislocation of knee NOS (*n* = 7,345; 1%)
Mechanical low back pain (*n* = 5,532; 2%)	Tietze’s disease (*n* = 1,790; 2%)	Toe pain (*n* = 27,652; 4%)	Ganglion of wrist (*n* = 13,804; 4%)	Painful right knee (*n* = 3,478; 1%)
Traumatic and/or non-traumatic injury of back (*n* = 4,542; 1%)	Acquired pectus carinatum (*n* = 1,647; 1%)	Injury of toe (*n* = 25,966; 4%)	Finger pain (*n* = 13,682; 4%)	Swollen knee (*n* = 3,314; 1%)
Pain in lumbar spine (*n* = 4,539; 1%)	Acquired pectus excavatum (*n* = 1,528; 1%)	Achilles tendinitis (*n* = 19,660; 3%)	Pain in thumb (*n* = 9,725; 3%)	Patellar tendinitis (*n* = 3,266; 1%)

* *C/O* Complaining of, *NOS* Not otherwise specified

There were age and gender differences in the top 5 codes for foot/ankle, hand/wrist and knee pain. Similar proportions of females with foot/ankle consultations across age bands were recorded with foot/ankle pain (24%−27%) or sprain/injury (24%−28%) whereas inverse trends were recorded for males with foot/ankle/heel pain (31% aged 8–11, 20% aged 12–15 and 19% aged 16–18) versus foot/ankle sprain/injury (17% aged 8–11, 27% aged 12–15 and 39% aged 16–18; Tables S3a-S3b).

The most common code for knee was “*Knee pain*” accounting for 42%−54% of records with differences observed by age and gender in the second to fourth codes (“*Knee injury*”; “*Juvenile osteochondrosis of tibial tubercle*”; “*Anterior knee pain*”); “*Juvenile osteochondrosis of tibial tubercle*” was the second most common code for those aged 12–15 years (21% males; 8% females) while “*Anterior knee pain*” was more common in females (5%−7% versus 4% for all age bands in males; Tables S3a-S3b).

There was little difference by age and gender for back pain with the top 3 codes (“*Low back pain*”; “*Back pain*”; “*C/O low back pain*”) accounting for 48% −56% records and the top 4 codes for chest/trunk pain (“*Musculoskeletal chest pain*”; “*Costochondritis*”; “*Rib pain*”; “*Chest wall pain*”) accounting for 74% −81% records (Tables S3a-S3b).

## Discussion

This study using a nationally representative primary care database of consultation records from over 4.1 million children and young people has shown that nearly one in ten children and young people seek healthcare each year for musculoskeletal pain. Consultation is higher in females than males in those aged 8–11; and higher consultation prevalence was seen in females and males aged 16–18 than those aged 8–11, or 12–15. Consultation in younger children is driven by foot and ankle pain. Whilst knee pain becomes more common in older children, gender variation is seen with the predominance of back pain in older females. There is some evidence that sprain/injury codes are more frequently used in males. The data presented here will inform and underpin future research on the management of musculoskeletal pain in children and young people, an area where there is a substantial evidence gap [14]. 

### Comparison to other studies

This study has shown that around one in ten children and young people aged 8–18 present to primary care with musculoskeletal pain each year; with lowest consultation in children aged 8–11. Our results add detail to previous findings using local (North Staffordshire) data which demonstrated that from 5% of 6–9 year-olds to 12% of 14–17 year-olds visit their General Practitioner annually for musculoskeletal problems [15]. Similarly, Canadian research found 12% of children and young people sought healthcare for musculoskeletal disorders (74% from primary care) [16]. A recent study from Sweden reported that over 12-months, 2–3% of 13–18 year olds presented with back/neck pain with 80% occurring in primary care, again consistent with our figure of 118/10,000 in 2019 for back pain in those aged 8–18 [17]. Our research also showed that children are more likely to seek healthcare for musculoskeletal consultations as they become older [15], confirming findings from Australia and Sweden [17, 18]. By using this large dataset, we have been able to show that this increase with age is not present for all pain sites, and differs by gender, which was also shown by a previous study in Australian primary care covering the period 2006–2011 [18]. The distinct rise in healthcare seeking for back pain among older girls has not previously been demonstrated and warrants further investigation, as it may give insights into the development of back pain, which is the leading cause of disability globally among adults [1, 19]. One potential explanation might be menstrual cramps or pain that may present as or co-present with lower back pain [20], and requires further exploration in primary care.

The fall in consultation prevalence and incidence in 2020 at the time of the COVID-19 pandemic, and the gradual rise in 2021 matches patterns of musculoskeletal consultation found in all age populations, with fewer individuals consulting UK primary care for musculoskeletal pain conditions, including inflammatory arthritis [12, 13, 21]. This is likely to reflect reduced care seeking behaviour and future research should assess the impact on long term musculoskeletal outcomes of reductions in seeking care during this period.

Williams et al. (2022) demonstrated that foot, ankle, and leg problems are a common reason for children to consult primary care in Australia, with consultation rates increasing with age and peaking in adolescence [22]. Their findings support our observation that foot/ankle pain is the most frequent site of musculoskeletal consultation in younger children, highlighting the importance of age-specific patterns in healthcare seeking for musculoskeletal pain. However, the predominance of foot and ankle consultation found in younger children does not match patterns in adults where back pain is generally reported to have the highest prevalence followed by the knee [2]. However, a previous evidence synthesis of epidemiological studies observed a gradual decline in the incidence of ankle sprains as people move through younger ages into adulthood and the authors’ postulated that this trend may relate to motor control development [23]. Our observation of increased sprain/injury codes in males may reflect trends of higher physical activity levels among young males compared with young females [24]. Although largely speculative, other explanations for the high foot and ankle related consultation patterns in younger ages may include musculoskeletal growth (for example, calcaneal apophysitis in the heel [25]) or possibly relate to concerns about flatter foot profiles [26]. More broadly, during formative years, contextual factors such as the family structure may influence how young people experience and seek care for musculoskeletal pain [27]. 

### Strengths and limitations

There has been little prior evidence of national consultation prevalence and incidence patterns for musculoskeletal pain in children and young people in primary care. CPRD Aurum is a large national primary care database, broadly representative of UK primary care with ~ 20% national coverage [10], although 99% of general practices are located in England. This large sample size has allowed us to present data by individual ages, genders and pain sites, uncovering previously unseen patterns.

Given that there may be impacts of hormonal changes during adolescence that are related to biological sex, it is worth noting that individuals can request to have their gender identity recorded instead of biological sex on their primary care record (following *Gender Recognition Act 2004* [28]), although this is likely to affect a minority of individuals consulting (incident gender dysmorphia: 4.4 per 10,000 person years in 2021 for 0–18 year-olds [29]).

Our definition of incident musculoskeletal pain was informed by consultation with our patient and public advisory group, and so rather than attempting to describe true incident (first ever) consultations for musculoskeletal pain, we defined a gap of 12 months between consultations for musculoskeletal pain as a new episode.

It is possible we have introduced a selection bias by excluding children and young people who do not have 2 years prior registration at their general practice. Residential mobility is associated with economic instability and family status [30], and might impact on healthcare utilisation and record completion. However, it is not possible to explore the impact of these factors within routinely collected primary care data in the UK.

## Conclusion

Annual consultation prevalence and incidence rates for musculoskeletal pain in children and young people were stable pre-pandemic, although key differences were observed by age, gender, and pain site being consulted for. Understanding the patterns of musculoskeletal pain in children and young people presenting to primary care is key, due to paucity of information on how to effectively treat and care for this population. Future work will explore consultations for rarer musculoskeletal conditions, short-term management of incident musculoskeletal pain and longer-term prognosis in those consulting for non-traumatic musculoskeletal pain, and costs associated with primary health care.

## Supplementary Information

Supplementary Material 1.

## Data Availability

Data may be obtained from a third party and are not publicly available. The data were obtained from the Clinical Practice Research Datalink. Clinical Practice Research Datalink data governance does not allow us to distribute patient data to other parties. Researchers may apply for data access at [http://www.CPRD.com/](http:/www.CPRD.com).

## Abbreviations

C/O: Complaining of
COVID-19: Coronavirus disease 2019
CPRD: Clinical Practice Research Datalink
EMIS: Egton Medical Information Systems
IQR: Interquartile range
NHS: National Health Service
NICE: National Institute for Health and Care Excellence
NOS: Not otherwise specified
SNOMED CT: Systematized Nomenclature of Medicine Clinical Terms
