# Supplementary material for: Trends of musculoskeletal pain in children and young people consulting primary care: an electronic primary health care record study
Source: BMC Pediatr. 2025 Nov 25;25:961. doi: 10.1186/s12887-025-06296-y (PMC12648837; doi:10.1186/s12887-025-06296-y)
Supplement: Supplementary file 1 — Supplementary Material 1. [file 12887_2025_6296_MOESM1_ESM.docx]

**Supplementary Materials**

Fig. S1 - Annual incidence rates of musculoskeletal consultations by age and gender per 10,000 registered population

Fig. S2 - Annual incidence rates of the most common regional pain sites per 10,000 registered population

Fig. S3 - Annual incidence of pain sites by age and gender per 10,000 registered population

Fig. S4 - Annual prevalence of pain sites by age and gender per 10,000 registered population

Table S1a - Annual incidence of musculoskeletal consultations per 10,000 registered population by age and site

Table S1b - Annual incidence of musculoskeletal consultations per 10,000 registered population by age and site in females

Table S1c - Annual incidence of musculoskeletal consultations per 10,000 registered population by age and site in males

Table S2a - Annual prevalence of musculoskeletal consultations per 10,000 registered population by age and site

Table S2b - Annual prevalence of musculoskeletal consultations per 10,000 registered population by age and site in females

Table S2c - Annual prevalence of musculoskeletal consultations per 10,000 registered population by age and site in males

Table S3a - Top 5 codes for Back, Knee, Foot/Ankle, Hand/Wrist and Chest/Trunk by age in Females

Table S3b - Top 5 codes for Back, Knee, Foot/Ankle, Hand/Wrist and Chest/Trunk by age in Males


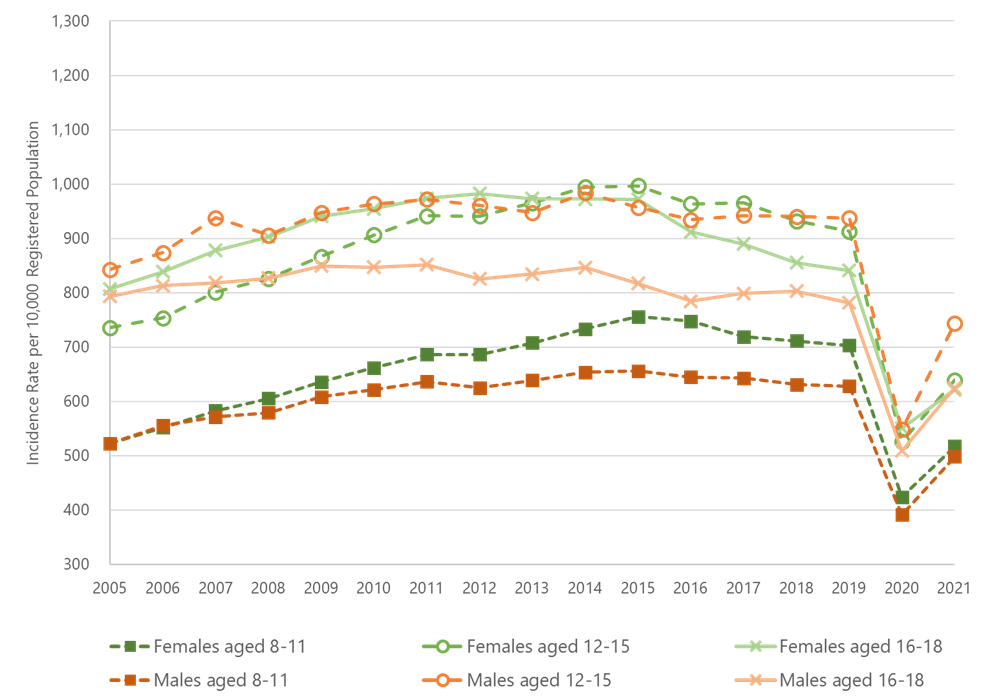


**Fig. S1** The image depicts annual incidence rates of musculoskeletal consultations between 2005-2021 per 10,000 registered population. The rates are presented by the age groups 8-11 years (square marker, dotted lines), 12-15 years (circle marker, dashed lines) and 16-18 years (cross marker, solid lines) and by gender (females in green; males in orange).


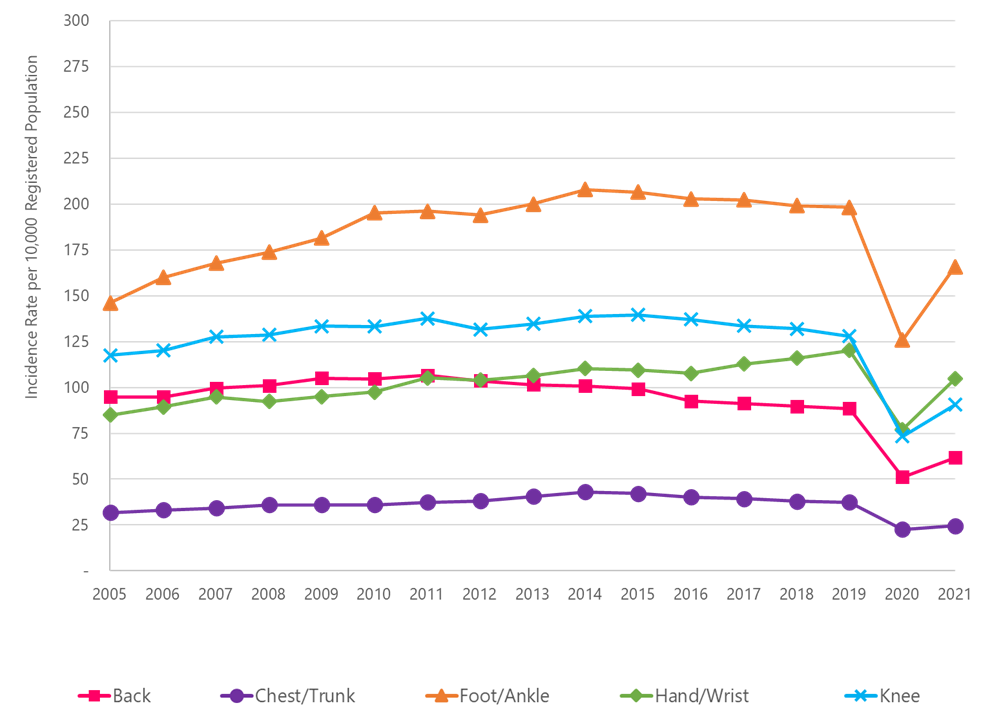


**Fig. S2** The image depicts annual incidence rates of the most common regional pain sites per 10,000 registered population between 2005-2021. The rates are presented for back pain (square marker, pink line), chest/trunk pain (circle marker, purple line), foot/ankle pain (triangle marker, orange line), hand/wrist pain (diamond marker, green line) and knee pain (cross marker, blue line).


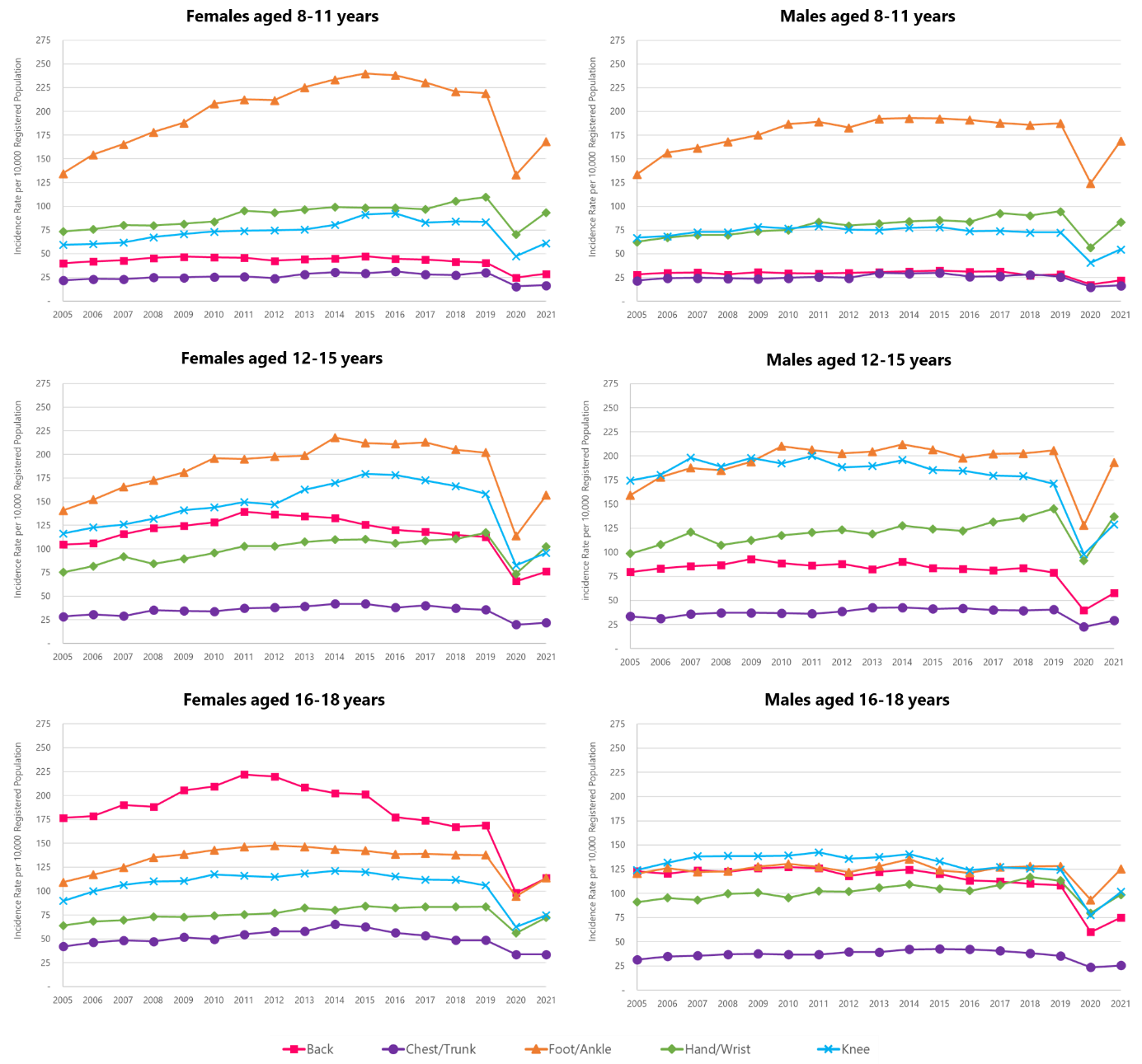


**Fig. S3** The image depicts 6 graphs with annual incidence rates of the most common regional pain sites by age and gender per 10,000 registered population between 2005-2021. Graphs for females are on the left and graphs for males on the right. Ages 8-11 are presented on the top row, followed by ages 12-15 then ages 16-18. The rates are presented for back pain (square marker, pink line), chest/trunk pain (circle marker, purple line), foot/ankle pain (triangle marker, orange line), hand/wrist pain (diamond marker, green line) and knee pain (cross marker, blue line).


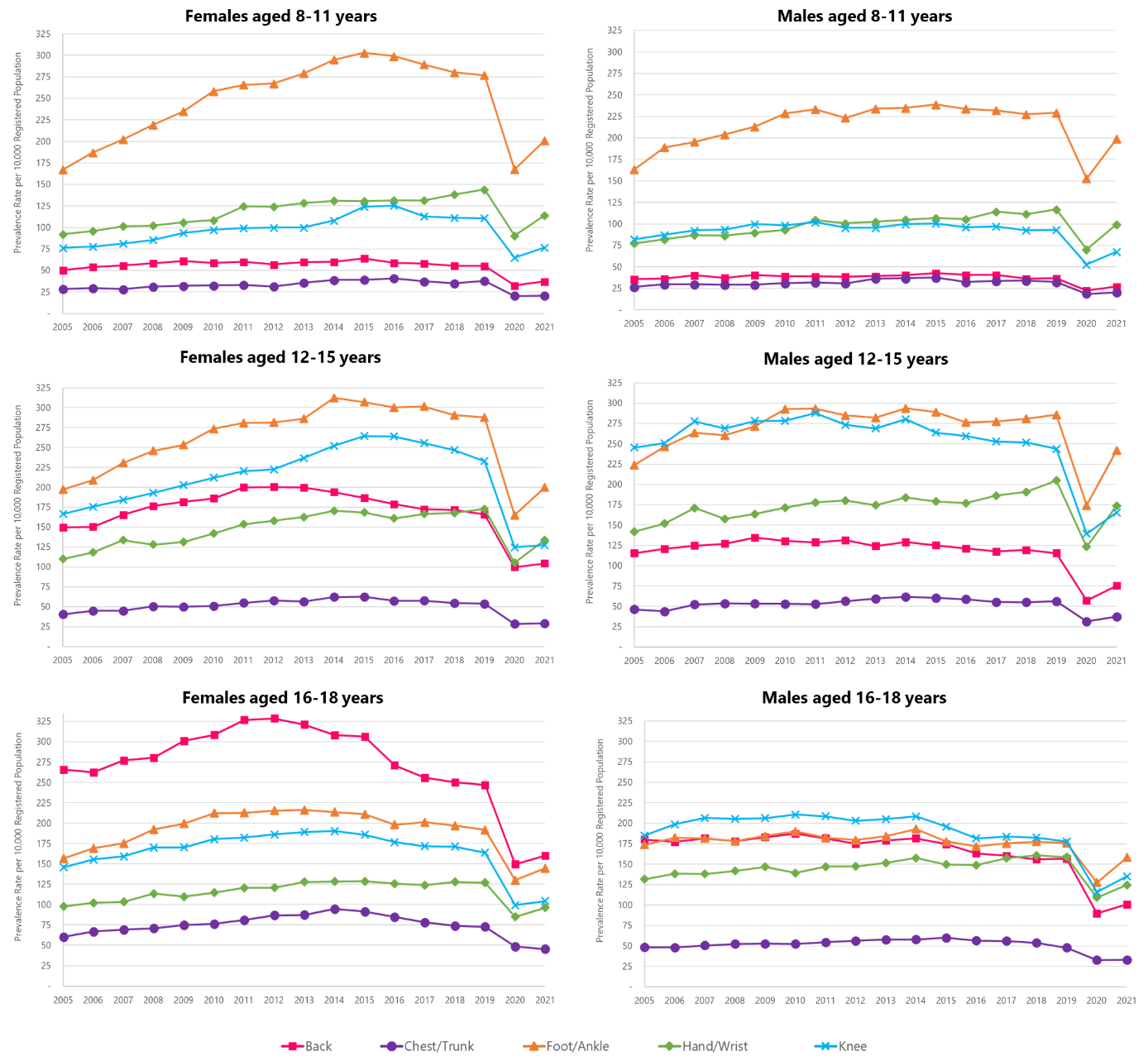


**Fig. S4** The image depicts 6 graphs with annual prevalence rates of the most common regional pain sites by age and gender per 10,000 registered population between 2005-2021. Graphs for females are on the left and graphs for males on the right. Ages 8-11 are presented on the top row, followed by ages 12-15 then ages 16-18. The rates are presented for back pain (square marker, pink line), chest/trunk pain (circle marker, purple line), foot/ankle pain (triangle marker, orange line), hand/wrist pain (diamond marker, green line) and knee pain (cross marker, blue line).

Table S1a - Annual Incidence of musculoskeletal consultations per 10,000 registered population by age and site

|  |  | Age Bands |  |  | Pain Sites |  |  |  |  |
| --- | --- | --- | --- | --- | --- | --- | --- | --- | --- |
| Year | Total | 8-11 years | 12-15 years | 16-18 years | Back | Chest/Trunk | Foot/Ankle | Hand/Wrist | Knee |
| 2005 | 699 (694, 704) | 523 (516, 530) | 794 (785, 802) | 800 (790, 810) | 95 (93, 97) | 32 (31, 33) | 146 (144, 148) | 85 (83, 87) | 117 (115, 119) |
| 2006 | 726 (721, 731) | 554 (546, 561) | 819 (810, 827) | 825 (814, 835) | 95 (93, 97) | 33 (32, 34) | 160 (158, 162) | 89 (88, 91) | 120 (118, 122) |
| 2007 | 760 (755, 765) | 577 (569, 584) | 875 (866, 884) | 846 (835, 856) | 100 (98, 101) | 34 (33, 35) | 168 (165, 170) | 95 (93, 97) | 128 (126, 130) |
| 2008 | 767 (762, 772) | 591 (584, 599) | 869 (860, 878) | 861 (851, 871) | 101 (99, 103) | 36 (35, 37) | 174 (171, 176) | 92 (91, 94) | 129 (126, 131) |
| 2009 | 800 (794, 805) | 621 (614, 629) | 910 (901, 919) | 891 (880, 901) | 105 (103, 107) | 36 (35, 37) | 182 (179, 184) | 95 (93, 97) | 133 (131, 135) |
| 2010 | 816 (811, 821) | 641 (633, 648) | 937 (928, 947) | 896 (885, 906) | 105 (103, 107) | 36 (35, 37) | 195 (193, 198) | 97 (96, 99) | 133 (131, 135) |
| 2011 | 832 (827, 837) | 660 (653, 668) | 958 (948, 967) | 907 (897, 918) | 107 (105, 109) | 37 (36, 38) | 196 (194, 199) | 105 (103, 107) | 138 (135, 140) |
| 2012 | 823 (817, 828) | 655 (647, 662) | 952 (942, 961) | 897 (887, 908) | 104 (102, 106) | 38 (37, 39) | 194 (192, 197) | 104 (102, 106) | 132 (130, 134) |
| 2013 | 829 (824, 835) | 672 (665, 680) | 956 (947, 965) | 898 (887, 908) | 102 (100, 103) | 41 (39, 42) | 200 (197, 202) | 107 (105, 108) | 135 (133, 137) |
| 2014 | 849 (844, 854) | 693 (685, 700) | 989 (980, 998) | 904 (893, 914) | 101 (99, 103) | 43 (42, 44) | 208 (205, 210) | 110 (108, 112) | 139 (137, 141) |
| 2015 | 846 (841, 851) | 705 (698, 712) | 976 (967, 985) | 888 (878, 898) | 99 (97, 101) | 42 (41, 43) | 207 (204, 209) | 109 (108, 111) | 140 (138, 142) |
| 2016 | 822 (817, 827) | 695 (688, 702) | 948 (940, 957) | 843 (834, 853) | 93 (91, 94) | 40 (39, 41) | 203 (200, 205) | 108 (106, 109) | 137 (135, 139) |
| 2017 | 818 (813, 823) | 680 (673, 687) | 954 (945, 962) | 841 (831, 851) | 91 (90, 93) | 39 (38, 40) | 202 (200, 205) | 113 (111, 115) | 134 (132, 136) |
| 2018 | 806 (801, 810) | 670 (664, 677) | 936 (928, 944) | 827 (818, 837) | 90 (88, 91) | 38 (37, 39) | 199 (197, 201) | 116 (114, 118) | 132 (130, 134) |
| 2019 | 796 (792, 801) | 665 (658, 671) | 925 (917, 933) | 810 (801, 819) | 88 (87, 90) | 37 (36, 38) | 198 (196, 201) | 120 (119, 122) | 128 (126, 130) |
| 2020 | 486 (482, 489) | 407 (402, 413) | 537 (531, 543) | 529 (522, 536) | 51 (50, 52) | 23 (22, 23) | 126 (124, 128) | 77 (76, 78) | 73 (72, 75) |
| 2021 | 606 (602, 610) | 508 (502, 513) | 693 (686, 700) | 623 (616, 631) | 62 (60, 63) | 25 (24, 25) | 166 (164, 168) | 105 (103, 106) | 91 (89, 92) |

Table S1b - Annual Incidence of musculoskeletal consultations per 10,000 registered population by age and site in females

|  |  | Age Bands |  |  | Pain Sites |  |  |  |  |
| --- | --- | --- | --- | --- | --- | --- | --- | --- | --- |
| Year | Females | 8-11 years | 12-15 years | 16-18 years | Back | Chest/Trunk | Foot/Ankle | Hand/Wrist | Knee |
| 2005 | 678 (671, 685) | 523 (512, 533) | 736 (723, 748) | 807 (792, 822) | 101 (98, 104) | 30 (28, 31) | 130 (127, 133) | 71 (69, 74) | 89 (86, 91) |
| 2006 | 704 (697, 712) | 552 (541, 562) | 753 (741, 766) | 838 (823, 854) | 103 (100, 106) | 32 (31, 34) | 143 (140, 146) | 76 (73, 78) | 94 (91, 97) |
| 2007 | 743 (736, 751) | 583 (572, 594) | 801 (789, 814) | 878 (862, 893) | 110 (107, 113) | 32 (31, 34) | 154 (151, 158) | 81 (79, 84) | 97 (95, 100) |
| 2008 | 767 (759, 774) | 605 (594, 616) | 826 (813, 839) | 903 (887, 919) | 113 (110, 115) | 35 (33, 36) | 164 (161, 168) | 79 (77, 82) | 102 (100, 105) |
| 2009 | 801 (794, 809) | 635 (624, 647) | 867 (853, 880) | 941 (925, 957) | 118 (115, 121) | 36 (34, 37) | 172 (168, 175) | 82 (79, 84) | 107 (104, 109) |
| 2010 | 827 (819, 835) | 662 (651, 673) | 906 (893, 920) | 954 (938, 970) | 119 (116, 122) | 35 (33, 37) | 186 (183, 190) | 85 (83, 88) | 110 (107, 113) |
| 2011 | 851 (843, 859) | 686 (675, 697) | 941 (928, 955) | 973 (957, 990) | 125 (122, 127) | 37 (36, 39) | 189 (185, 193) | 93 (90, 95) | 112 (109, 114) |
| 2012 | 850 (843, 858) | 687 (676, 697) | 941 (927, 954) | 983 (966, 999) | 120 (117, 123) | 38 (36, 39) | 190 (187, 194) | 92 (90, 95) | 110 (107, 113) |
| 2013 | 863 (855, 870) | 708 (697, 719) | 965 (952, 979) | 973 (957, 989) | 116 (114, 119) | 40 (38, 41) | 196 (193, 200) | 97 (94, 99) | 116 (114, 119) |
| 2014 | 882 (874, 889) | 733 (723, 744) | 995 (981, 1008) | 973 (956, 989) | 113 (110, 116) | 43 (41, 45) | 206 (203, 210) | 98 (96, 101) | 121 (118, 124) |
| 2015 | 892 (885, 899) | 756 (745, 767) | 997 (984, 1010) | 972 (956, 988) | 111 (109, 114) | 42 (40, 43) | 207 (203, 211) | 99 (97, 102) | 129 (126, 132) |
| 2016 | 864 (857, 871) | 748 (737, 758) | 963 (951, 976) | 912 (897, 927) | 103 (100, 105) | 40 (38, 41) | 205 (202, 209) | 97 (95, 100) | 129 (126, 131) |
| 2017 | 849 (842, 856) | 719 (709, 729) | 965 (953, 978) | 890 (875, 904) | 101 (99, 104) | 39 (37, 40) | 203 (199, 206) | 98 (96, 100) | 122 (120, 125) |
| 2018 | 827 (820, 833) | 712 (702, 721) | 932 (920, 943) | 855 (841, 869) | 98 (96, 101) | 36 (35, 37) | 195 (192, 199) | 102 (100, 104) | 121 (119, 124) |
| 2019 | 814 (807, 821) | 703 (693, 713) | 913 (901, 924) | 841 (828, 855) | 98 (96, 101) | 37 (35, 38) | 193 (190, 196) | 106 (104, 108) | 116 (114, 119) |
| 2020 | 493 (488, 499) | 424 (416, 432) | 526 (517, 535) | 551 (540, 562) | 58 (57, 60) | 22 (21, 23) | 116 (114, 119) | 68 (66, 70) | 64 (62, 66) |
| 2021 | 589 (584, 595) | 517 (509, 526) | 639 (630, 649) | 622 (611, 633) | 68 (66, 70) | 23 (22, 24) | 150 (147, 152) | 91 (89, 93) | 77 (75, 79) |

Table S1c - Annual Incidence of musculoskeletal consultations per 10,000 registered population by age and site in males

|  |  | Age Bands |  |  | Pain Sites |  |  |  |  |
| --- | --- | --- | --- | --- | --- | --- | --- | --- | --- |
| Year | Males | 8-11 years | 12-15 years | 16-18 years | Back | Chest/Trunk | Foot/Ankle | Hand/Wrist | Knee |
| 2005 | 716 (709, 723) | 523 (513, 533) | 843 (831, 855) | 794 (780, 807) | 73 (71, 76) | 29 (28, 30) | 139 (136, 142) | 84 (81, 86) | 123 (120, 125) |
| 2006 | 744 (738, 751) | 556 (546, 566) | 874 (861, 886) | 813 (799, 827) | 75 (73, 77) | 30 (28, 31) | 156 (153, 159) | 90 (88, 92) | 127 (124, 130) |
| 2007 | 774 (767, 781) | 572 (562, 582) | 938 (925, 951) | 819 (805, 832) | 77 (75, 79) | 32 (30, 33) | 160 (157, 163) | 95 (92, 97) | 137 (134, 140) |
| 2008 | 767 (760, 774) | 579 (569, 589) | 906 (893, 918) | 827 (813, 840) | 76 (74, 78) | 33 (31, 34) | 161 (158, 165) | 92 (89, 94) | 133 (131, 136) |
| 2009 | 798 (791, 805) | 608 (598, 619) | 947 (935, 960) | 849 (835, 863) | 80 (78, 82) | 32 (31, 34) | 168 (165, 172) | 95 (93, 97) | 138 (135, 141) |
| 2010 | 807 (800, 814) | 621 (611, 631) | 964 (951, 976) | 847 (833, 860) | 78 (76, 80) | 32 (31, 34) | 179 (176, 183) | 96 (93, 98) | 135 (133, 138) |
| 2011 | 815 (808, 822) | 636 (626, 646) | 972 (959, 985) | 852 (838, 866) | 76 (74, 78) | 33 (31, 34) | 178 (175, 182) | 102 (99, 104) | 140 (137, 143) |
| 2012 | 798 (791, 805) | 625 (615, 635) | 961 (948, 973) | 826 (812, 839) | 74 (72, 76) | 34 (32, 35) | 174 (170, 177) | 101 (99, 104) | 132 (129, 135) |
| 2013 | 800 (793, 806) | 639 (629, 648) | 948 (935, 960) | 835 (821, 848) | 73 (71, 75) | 37 (35, 38) | 180 (176, 183) | 101 (99, 104) | 132 (129, 135) |
| 2014 | 819 (812, 826) | 653 (644, 663) | 984 (971, 997) | 846 (832, 860) | 76 (74, 78) | 37 (36, 39) | 185 (181, 188) | 106 (103, 108) | 135 (132, 138) |
| 2015 | 803 (796, 810) | 656 (646, 665) | 957 (945, 969) | 817 (804, 831) | 73 (71, 75) | 37 (36, 39) | 180 (177, 183) | 104 (102, 106) | 130 (127, 133) |
| 2016 | 783 (777, 790) | 645 (636, 654) | 934 (922, 946) | 785 (772, 798) | 70 (68, 72) | 36 (34, 37) | 176 (173, 179) | 102 (100, 104) | 126 (123, 129) |
| 2017 | 789 (783, 796) | 643 (634, 652) | 942 (931, 954) | 799 (786, 812) | 70 (68, 71) | 35 (34, 36) | 178 (175, 181) | 111 (108, 113) | 125 (123, 128) |
| 2018 | 786 (779, 792) | 631 (622, 640) | 940 (929, 952) | 803 (790, 815) | 68 (66, 70) | 35 (34, 36) | 178 (175, 181) | 113 (111, 116) | 124 (122, 127) |
| 2019 | 779 (773, 786) | 628 (619, 637) | 937 (926, 949) | 781 (769, 794) | 67 (65, 69) | 34 (32, 35) | 179 (176, 182) | 118 (115, 120) | 122 (119, 124) |
| 2020 | 479 (474, 484) | 391 (384, 399) | 548 (539, 557) | 509 (499, 519) | 36 (35, 38) | 20 (19, 21) | 118 (115, 120) | 75 (73, 77) | 71 (69, 73) |
| 2021 | 622 (616, 627) | 499 (490, 507) | 744 (734, 754) | 625 (614, 636) | 49 (47, 50) | 24 (23, 25) | 166 (164, 169) | 107 (105, 109) | 94 (92, 96) |

Table S2a - Annual Prevalence of musculoskeletal consultations per 10,000 registered population by age and site

|  |  | Age Bands | | | Pain Sites | | | | |
| --- | --- | --- | --- | --- | --- | --- | --- | --- | --- |
| Year | Total | 8-11 years | 12-15 years | 16-18 years | Back | Chest/Trunk | Foot/Ankle | Hand/Wrist | Knee |
| 2005 | 808 (803, 813) | 569 (562, 577) | 928 (919, 938) | 957 (946, 968) | 124 (122, 126) | 41 (39, 42) | 182 (180, 185) | 109 (107, 111) | 151 (149, 154) |
| 2006 | 843 (838, 849) | 604 (596, 612) | 960 (950, 969) | 997 (986, 1008) | 125 (123, 127) | 43 (41, 44) | 200 (197, 202) | 115 (113, 117) | 158 (156, 161) |
| 2007 | 888 (883, 894) | 632 (624, 640) | 1035 (1025, 1044) | 1026 (1015, 1037) | 132 (130, 134) | 45 (43, 46) | 211 (208, 214) | 123 (121, 125) | 168 (165, 170) |
| 2008 | 900 (894, 905) | 648 (640, 656) | 1038 (1028, 1048) | 1045 (1033, 1056) | 134 (132, 136) | 47 (45, 48) | 219 (216, 222) | 121 (119, 124) | 170 (167, 172) |
| 2009 | 936 (930, 941) | 682 (674, 690) | 1081 (1071, 1091) | 1081 (1070, 1093) | 140 (138, 142) | 47 (46, 48) | 229 (226, 231) | 125 (123, 127) | 175 (173, 178) |
| 2010 | 960 (955, 966) | 706 (698, 714) | 1119 (1109, 1129) | 1096 (1085, 1108) | 140 (138, 142) | 48 (46, 49) | 246 (243, 248) | 128 (126, 130) | 178 (176, 181) |
| 2011 | 980 (974, 986) | 732 (724, 740) | 1146 (1136, 1156) | 1110 (1098, 1121) | 142 (140, 144) | 49 (48, 50) | 248 (246, 251) | 138 (136, 140) | 182 (179, 184) |
| 2012 | 974 (968, 979) | 725 (718, 733) | 1148 (1138, 1158) | 1106 (1095, 1118) | 140 (138, 142) | 51 (49, 52) | 245 (242, 248) | 138 (136, 140) | 177 (174, 179) |
| 2013 | 977 (971, 982) | 742 (734, 749) | 1147 (1137, 1157) | 1103 (1092, 1115) | 138 (136, 140) | 53 (52, 54) | 251 (248, 254) | 140 (138, 142) | 178 (176, 180) |
| 2014 | 996 (991, 1002) | 765 (757, 772) | 1183 (1173, 1193) | 1107 (1095, 1118) | 135 (133, 137) | 56 (54, 57) | 262 (259, 265) | 145 (143, 147) | 185 (182, 187) |
| 2015 | 993 (987, 998) | 779 (772, 787) | 1168 (1158, 1178) | 1089 (1078, 1100) | 133 (131, 135) | 55 (54, 57) | 261 (258, 263) | 143 (141, 145) | 185 (183, 188) |
| 2016 | 964 (959, 970) | 770 (762, 777) | 1136 (1126, 1145) | 1032 (1021, 1043) | 125 (123, 127) | 52 (51, 54) | 254 (251, 256) | 141 (139, 143) | 181 (179, 184) |
| 2017 | 955 (950, 960) | 755 (747, 762) | 1134 (1125, 1144) | 1013 (1003, 1024) | 121 (119, 123) | 51 (49, 52) | 253 (250, 256) | 147 (145, 149) | 177 (174, 179) |
| 2018 | 941 (936, 946) | 741 (734, 748) | 1115 (1106, 1124) | 1003 (993, 1014) | 119 (118, 121) | 49 (48, 50) | 249 (246, 251) | 149 (147, 151) | 174 (172, 176) |
| 2019 | 931 (926, 936) | 735 (728, 742) | 1104 (1095, 1113) | 982 (972, 992) | 118 (117, 120) | 48 (47, 50) | 248 (245, 250) | 155 (153, 157) | 169 (167, 171) |
| 2020 | 580 (576, 584) | 456 (451, 462) | 656 (649, 663) | 656 (648, 664) | 69 (68, 70) | 29 (28, 29) | 155 (153, 157) | 97 (95, 98) | 98 (96, 100) |
| 2021 | 678 (674, 682) | 548 (542, 554) | 780 (773, 787) | 719 (710, 727) | 78 (77, 80) | 30 (29, 31) | 195 (193, 197) | 125 (123, 127) | 112 (110, 114) |

Table S2b - Annual Prevalence of musculoskeletal consultations per 10,000 registered population by age and site in females

|  |  | Age Bands | | | Pain Sites | | | | |
| --- | --- | --- | --- | --- | --- | --- | --- | --- | --- |
| Year | Females | 8-11 years | 12-15 years | 16-18 years | Back | Chest/Trunk | Foot/Ankle | Hand/Wrist | Knee |
| 2005 | 789 (782, 797) | 574 (563, 585) | 864 (850, 877) | 975 (958, 991) | 145 (142, 149) | 41 (40, 43) | 175 (172, 179) | 100 (97, 103) | 128 (125, 132) |
| 2006 | 823 (816, 831) | 605 (594, 616) | 891 (877, 904) | 1020 (1003, 1037) | 146 (143, 150) | 45 (44, 47) | 190 (186, 194) | 106 (103, 108) | 135 (132, 138) |
| 2007 | 869 (861, 877) | 639 (627, 650) | 953 (939, 967) | 1059 (1042, 1076) | 156 (153, 160) | 46 (44, 47) | 205 (201, 209) | 113 (110, 116) | 140 (137, 143) |
| 2008 | 903 (895, 911) | 666 (654, 677) | 989 (975, 1004) | 1102 (1085, 1120) | 162 (159, 166) | 49 (47, 51) | 221 (217, 225) | 114 (112, 117) | 147 (144, 151) |
| 2009 | 942 (934, 950) | 702 (690, 713) | 1033 (1019, 1048) | 1148 (1130, 1165) | 170 (166, 173) | 50 (48, 52) | 232 (227, 236) | 116 (113, 119) | 153 (150, 157) |
| 2010 | 977 (968, 985) | 733 (721, 745) | 1087 (1072, 1101) | 1175 (1157, 1192) | 171 (167, 174) | 51 (49, 53) | 251 (247, 255) | 122 (119, 125) | 160 (157, 164) |
| 2011 | 1010 (1002, 1019) | 766 (754, 777) | 1133 (1118, 1148) | 1208 (1190, 1226) | 178 (175, 182) | 53 (51, 55) | 257 (253, 261) | 133 (130, 136) | 163 (160, 167) |
| 2012 | 1014 (1006, 1023) | 766 (755, 778) | 1146 (1131, 1161) | 1223 (1205, 1241) | 176 (172, 179) | 55 (53, 57) | 259 (255, 263) | 135 (132, 138) | 164 (161, 168) |
| 2013 | 1025 (1017, 1034) | 788 (777, 800) | 1169 (1154, 1184) | 1212 (1194, 1230) | 173 (170, 176) | 56 (54, 58) | 266 (261, 270) | 140 (137, 143) | 169 (166, 173) |
| 2014 | 1048 (1040, 1056) | 817 (806, 829) | 1209 (1194, 1224) | 1211 (1193, 1229) | 166 (163, 169) | 60 (59, 62) | 281 (277, 285) | 144 (141, 147) | 178 (174, 181) |
| 2015 | 1056 (1048, 1064) | 842 (831, 853) | 1206 (1191, 1220) | 1204 (1186, 1221) | 164 (161, 168) | 60 (58, 62) | 282 (278, 287) | 143 (140, 146) | 188 (185, 192) |
| 2016 | 1027 (1019, 1034) | 836 (825, 847) | 1172 (1158, 1186) | 1134 (1117, 1151) | 151 (148, 154) | 57 (55, 59) | 276 (272, 280) | 141 (138, 143) | 187 (184, 191) |
| 2017 | 1003 (995, 1010) | 805 (795, 816) | 1166 (1153, 1179) | 1086 (1070, 1102) | 146 (143, 149) | 54 (52, 56) | 273 (269, 277) | 142 (140, 145) | 179 (176, 182) |
| 2018 | 980 (973, 987) | 793 (782, 803) | 1130 (1117, 1143) | 1059 (1043, 1074) | 144 (142, 147) | 52 (50, 53) | 264 (260, 268) | 147 (144, 149) | 175 (172, 179) |
| 2019 | 963 (956, 971) | 786 (775, 796) | 1104 (1091, 1116) | 1035 (1020, 1050) | 142 (140, 145) | 52 (51, 54) | 260 (256, 264) | 150 (147, 153) | 169 (166, 171) |
| 2020 | 599 (593, 605) | 481 (473, 490) | 658 (648, 668) | 690 (678, 702) | 87 (85, 89) | 30 (29, 32) | 157 (154, 160) | 94 (92, 97) | 95 (93, 98) |
| 2021 | 666 (661, 672) | 561 (552, 569) | 730 (720, 740) | 728 (716, 741) | 94 (91, 96) | 30 (29, 31) | 186 (183, 189) | 116 (114, 119) | 102 (100, 105) |

Table S2c - Annual Prevalence of musculoskeletal consultations per 10,000 registered population by age and site in males

|  |  | Age Bands | | | Pain Sites | | | | |
| --- | --- | --- | --- | --- | --- | --- | --- | --- | --- |
| Year | Males | 8-11 years | 12-15 years | 16-18 years | Back | Chest/Trunk | Foot/Ankle | Hand/Wrist | Knee |
| 2005 | 824 (817, 831) | 565 (555, 576) | 983 (970, 996) | 942 (927, 956) | 105 (103, 108) | 40 (38, 42) | 188 (185, 192) | 116 (113, 119) | 171 (168, 174) |
| 2006 | 860 (853, 868) | 603 (593, 614) | 1018 (1005, 1032) | 977 (962, 992) | 107 (104, 109) | 40 (38, 42) | 208 (204, 212) | 123 (120, 126) | 178 (175, 182) |
| 2007 | 905 (897, 912) | 627 (616, 637) | 1104 (1090, 1118) | 998 (983, 1013) | 111 (108, 113) | 44 (42, 46) | 216 (212, 220) | 132 (129, 135) | 192 (188, 195) |
| 2008 | 897 (890, 905) | 633 (622, 643) | 1080 (1066, 1093) | 997 (982, 1012) | 110 (107, 112) | 45 (43, 46) | 217 (213, 221) | 127 (125, 130) | 189 (185, 192) |
| 2009 | 931 (923, 938) | 664 (654, 675) | 1122 (1108, 1136) | 1026 (1011, 1041) | 114 (112, 117) | 45 (43, 46) | 226 (222, 230) | 132 (129, 135) | 194 (190, 197) |
| 2010 | 946 (938, 954) | 682 (671, 692) | 1147 (1133, 1161) | 1031 (1016, 1047) | 113 (110, 116) | 45 (43, 47) | 241 (237, 245) | 134 (131, 137) | 194 (191, 198) |
| 2011 | 954 (946, 961) | 702 (691, 713) | 1157 (1143, 1171) | 1027 (1012, 1043) | 110 (107, 113) | 46 (44, 47) | 241 (237, 245) | 142 (139, 145) | 198 (194, 201) |
| 2012 | 938 (930, 945) | 687 (676, 697) | 1150 (1136, 1164) | 1008 (993, 1023) | 108 (106, 111) | 47 (45, 49) | 233 (230, 237) | 141 (138, 144) | 188 (184, 191) |
| 2013 | 933 (925, 940) | 698 (687, 708) | 1128 (1114, 1142) | 1011 (996, 1026) | 106 (104, 109) | 50 (49, 52) | 238 (234, 241) | 141 (138, 144) | 186 (182, 189) |
| 2014 | 950 (942, 957) | 714 (704, 724) | 1160 (1146, 1174) | 1019 (1004, 1035) | 108 (105, 110) | 51 (49, 53) | 245 (241, 248) | 146 (143, 149) | 191 (188, 195) |
| 2015 | 935 (928, 942) | 720 (709, 730) | 1133 (1120, 1146) | 992 (977, 1007) | 105 (103, 108) | 51 (50, 53) | 241 (237, 244) | 143 (140, 146) | 182 (179, 186) |
| 2016 | 907 (900, 914) | 707 (697, 716) | 1102 (1089, 1115) | 944 (930, 958) | 100 (98, 102) | 48 (46, 50) | 233 (230, 237) | 142 (139, 144) | 176 (173, 179) |
| 2017 | 910 (903, 917) | 706 (697, 716) | 1104 (1092, 1117) | 950 (936, 964) | 98 (96, 100) | 47 (46, 49) | 234 (231, 238) | 151 (148, 153) | 175 (172, 178) |
| 2018 | 904 (898, 911) | 691 (682, 701) | 1100 (1088, 1112) | 953 (939, 967) | 96 (94, 98) | 47 (45, 48) | 234 (231, 238) | 152 (149, 155) | 172 (169, 175) |
| 2019 | 901 (894, 908) | 686 (677, 696) | 1105 (1092, 1117) | 934 (920, 947) | 95 (93, 98) | 45 (43, 46) | 236 (233, 240) | 159 (156, 162) | 169 (166, 172) |
| 2020 | 563 (557, 568) | 433 (425, 440) | 654 (645, 664) | 624 (613, 635) | 52 (51, 54) | 27 (26, 28) | 154 (151, 157) | 99 (97, 102) | 101 (98, 103) |
| 2021 | 689 (683, 695) | 537 (528, 545) | 828 (817, 838) | 709 (698, 721) | 64 (62, 66) | 30 (29, 31) | 204 (201, 207) | 133 (130, 136) | 121 (119, 123) |

Table S3a - Top 5 codes for Back, Knee, Foot/Ankle, Hand/Wrist and Chest/Trunk by age in females

| **Body Site** |  | **Aged 8-11 years** |  | **Aged 12-15 years** |  | **Aged 16-18 years** |
| --- | --- | --- | --- | --- | --- | --- |
| **Back** | 25% | Low back pain | 31% | Low back pain | 37% | Low back pain |
|  | 18% | Back pain | 12% | Back pain | 11% | Back pain |
|  | 5% | C/O - low back pain | 6% | C/O - low back pain | 8% | C/O - low back pain |
|  | 3% | Acute thoracic back pain | 4% | Coccygodynia | 2% | Mechanical low back pain |
|  | 2% | Coccygodynia | 3% | Idiopathic scoliosis | 1% | Coccygodynia |
| **Chest/Trunk** | 46% | Musculoskeletal chest pain | 36% | Musculoskeletal chest pain | 36% | Musculoskeletal chest pain |
|  | 16% | Costochondritis | 22% | Costochondritis | 23% | Costochondritis |
|  | 11% | Rib pain | 15% | Rib pain | 14% | Rib pain |
|  | 9% | Chest wall pain | 8% | Chest wall pain | 7% | Chest wall pain |
|  | 4% | Anterior chest wall pain | 3% | Anterior chest wall pain | 3% | Anterior chest wall pain |
| **Foot/Ankle** | 16% | Foot pain | 14% | Ankle sprain | 15% | Foot pain |
|  | 11% | Ankle sprain | 14% | Foot pain | 14% | Ankle sprain |
|  | 11% | Ankle pain | 10% | Ankle pain | 9% | Ankle pain |
|  | 7% | Foot injury | 8% | Ankle injury | 7% | Ankle injury |
|  | 6% | Ankle injury | 6% | Foot injury | 6% | Foot injury |
| **Hand/Wrist** | 12% | Wrist injury | 10% | Finger injury | 11% | Wrist joint pain |
|  | 10% | Finger injury | 8% | Wrist injury | 9% | Ganglion of wrist |
|  | 6% | Wrist sprain NOS | 8% | Wrist joint pain | 6% | Hand pain |
|  | 6% | Injury of hand | 7% | Injury of hand | 6% | Wrist injury |
|  | 5% | Wrist joint pain | 4% | Wrist sprain NOS | 5% | Finger injury |
| **Knee** | 52% | Knee pain | 52% | Knee pain | 54% | Knee pain |
|  | 11% | Knee injury | 8% | Juvenile osteochondrosis of tibial tubercle | 8% | Knee injury |
|  | 9% | Juvenile osteochondrosis of tibial tubercle | 7% | Anterior knee pain | 7% | Anterior knee pain |
|  | 5% | Anterior knee pain | 7% | Knee injury | 4% | Chondromalacia patellae |
|  | 2% | Knee sprain | 4% | Chondromalacia patellae | 2% | Knee sprain |

Table S3b - Top 5 codes for Back, Knee, Foot/Ankle, Hand/Wrist and Chest/Trunk by age in males

| **Body Site** |  | **Aged 8-11 years** |  | **Aged 12-15 years** |  | **Aged 16-18 years** |
| --- | --- | --- | --- | --- | --- | --- |
| **Back** | 25% | Low back pain | 32% | Low back pain | 36% | Low back pain |
|  | 19% | Back pain | 14% | Back pain | 11% | Back pain |
|  | 5% | C/O - low back pain | 6% | C/O - low back pain | 8% | C/O - low back pain |
|  | 4% | Traumatic and/or non-traumatic injury of back | 3% | Acute thoracic back pain | 2% | Acute thoracic back pain |
|  | 3% | Acute thoracic back pain | 2% | Coccygodynia | 2% | Mechanical low back pain |
| **Chest/Trunk** | 48% | Musculoskeletal chest pain | 40% | Musculoskeletal chest pain | 43% | Musculoskeletal chest pain |
|  | 12% | Costochondritis | 14% | Rib pain | 14% | Costochondritis |
|  | 12% | Rib pain | 12% | Costochondritis | 13% | Rib pain |
|  | 9% | Chest wall pain | 8% | Chest wall pain | 7% | Chest wall pain |
|  | 4% | Anterior chest wall pain | 3% | Symptom: chest wall | 3% | C/O - a chest wall symptom |
| **Foot/Ankle** | 13% | Foot pain | 12% | Foot pain | 19% | Ankle sprain |
|  | 10% | Heel pain | 11% | Ankle sprain | 12% | Ankle injury |
|  | 10% | Ankle sprain | 8% | Ankle pain | 10% | Foot pain |
|  | 8% | Ankle pain | 8% | Ankle injury | 9% | Ankle pain |
|  | 7% | Foot injury | 8% | Foot injury | 8% | Foot injury |
| **Hand/Wrist** | 13% | Finger injury | 10% | Finger injury | 13% | Injury of hand |
|  | 10% | Wrist injury | 10% | Injury of hand | 7% | Wrist joint pain |
|  | 8% | Thumb injury | 10% | Wrist injury | 7% | Finger injury |
|  | 7% | Injury of hand | 7% | Thumb injury | 7% | Wrist injury |
|  | 4% | Wrist sprain NOS | 4% | Wrist sprain NOS | 5% | Hand pain |
| **Knee** | 52% | Knee pain | 42% | Knee pain | 47% | Knee pain |
|  | 16% | Knee injury | 21% | Juvenile osteochondrosis of tibial tubercle | 14% | Knee injury |
|  | 7% | Juvenile osteochondrosis of tibial tubercle | 11% | Knee injury | 5% | Juvenile osteochondrosis of tibial tubercle |
|  | 4% | Anterior knee pain | 4% | Anterior knee pain | 4% | Anterior knee pain |
|  | 3% | Knee sprain | 2% | Knee sprain | 3% | Knee sprain |
